# Exploration of Public Perceptions of Wastewater Surveillance, United States, June 2024

**DOI:** 10.3201/eid3213.251494

**Published:** 2026-08

**Authors:** Jazmyn T. Moore, Krysten Rose-McCully, Diana Valencia, Hannah Turner, Kathleen Tatti, Manpreet Kaur, Mariatou Ceesay, Scott Santibanez

**Affiliations:** Centers for Disease Control and Prevention, Atlanta, Georgia, USA (J.T. Moore, K. Rose-McCully, D. Valencia, M. Kaur, M. Ceesay, S. Santibanez); Oak Ridge Institute for Science and Education, Oak Ridge, Tennessee, USA (K. Rose-McCully, M. Ceesay); Goldbelt Professional Services, Juneau, Alaska, USA (H. Turner); G^2^S Corporation, Shavano Park, Texas, USA (K. Tatti); Kennedy Krieger Institute, Dr. James A. Ferguson Emerging Infectious Diseases Graduate Fellowship, Baltimore, Maryland, USA (M. Kaur)

**Keywords:** wastewater surveillance, COVID-19, viruses, wastewater, infectious diseases, community engagement, surveillance, United States

## Abstract

Community wastewater surveillance (WS) is a valuable tool for informing public health action, but its effects on individual health behaviors rely on public understanding and awareness. We analyzed 2,024 free text responses from a national survey on perceptions of WS conducted during June 19–23, 2024, to gain insights into public perceptions of WS. Primary themes among 1,181 detailed responses included varying levels of trust in government and authorities, the need for more information about the effectiveness and accuracy of monitoring, desire to understand public health implications, and the need for more education and communication. Those findings could help inform community engagement strategies as WS continues to evolve.

In September 2020, the Centers for Disease Control and Prevention (CDC) launched the National Wastewater Surveillance System (NWSS) to monitor COVID-19 trends in wastewater samples across the United States (*1*). Wastewater surveillance (WS) has since expanded to include monitoring for influenza A, measles, and mpox viruses, as well as other pathogens, across ≈1,500 sites nationwide, including US states, territories, and tribal communities.

Surveillance of infectious diseases in wastewater provides communities with early warning signs of potential outbreaks and information about trends in infections. WS can inform individual protective behaviors and public health actions. A 2024 survey suggested that WS-specific awareness and education can enhance community trust in public health initiatives and motivate persons to take steps to protect themselves (*2*). Without continued efforts to educate and engage communities during the implementation of WS, implementers risk missteps that can harm trust in government and public health. We conducted a qualitative analysis of public perspectives on WS to help inform efforts to engage persons living in communities implementing WS.

## Methods

During June 19–23, 2024, Porter Novelli Public Services conducted a 9-question, nationwide survey among US adults (Appendix). The survey aimed to assess participants’ knowledge and attitudes regarding WS, referred to as wastewater monitoring in the survey (Appendix). In this qualitative analysis, we reviewed and categorized 2,024 free text responses to the open-ended question, “Do you have any concerns about wastewater monitoring in your community? Why or why not?”

Three analysts independently coded survey responses and subsequently reached consensus on themes. Each response was classified into the most relevant theme on the basis of its content. We calculated summary statistics to provide an overview of the findings. This activity was reviewed by members of the Information Collections and Human Studies Team in CDC’s National Center for Emerging and Zoonotic Infectious Diseases and was determined to be nonresearch in accordance with 45 CFR 46.102(l) on April 11, 2023. The survey was conducted in a manner consistent with applicable federal law and CDC policy.

## Results

Of 2,024 write-in responses, a total of 1,342 (66.3%) participants reported no concerns with WS, 285 (14.1%) expressed concerns, 248 (12.3%) were uncertain or did not understand the question, 110 (5.4%) were unaware of WS initiatives, and 39 (1.9%) relied on private wastewater systems only. From 1,181 responses, we identified the following 4 themes: trust in government and authorities, effectiveness and accuracy of monitoring, public health implications, education and communication (Table). Many respondents expressed multiple themes. For example, among 1,342 participants who expressed no concerns, 49% expressed no concerns at all, 43% supported the initiative with conditional trust in government agencies, and 6% expressed no concerns due to their unfamiliarity with the subject.

**Table:** Key themes from write-in responses describing perceptions and concerns around wastewater monitoring (n = 1,181). Porter Novelli Survey, U.S., June 2024

Theme	Sample quotes	Subthemes
Trust in government and authorities	“I trust the local health department to handle this. They’ve always been transparent and seem to genuinely care about our community’s well-being.”	• Past success during COVID-19 boosted trust in implementation • Concerns about nonhealth use of data • Negative experiences shaped federal government distrust • Fear that monitoring could stigmatize specific communities • Privacy was a key issue, with fears of being monitored
	“It sounds like something that could be helpful, but only if it’s done with complete honesty. Otherwise, I worry it’s just another way to monitor communities without telling us the full story.”	
Effectiveness and accuracy of monitoring	“There’s a lot of potential in this kind of monitoring, but I’d want to be sure the testing is consistent, and the results aren’t being manipulated or misunderstood. Public trust depends on transparency and good science.”	• Allows for real-time or near real-time surveillance • Seen as helpful in tracking patterns over time​ • Doubts about data accuracy and potential for skewed results • Concerns about misinterpretation or false alarms • Desire for more transparency on data handling and use
	“My main concern is wondering if the data is accurate or not and is quite possibly skewed by outside sources.”	
Public health implications	“As long as it’s used for the right reasons—like tracking disease and helping the community—then I fully support it. It’s a smart way to stay ahead of public health threats.”	• Seen as useful for early disease detection​ • Strong support when focused on public health​ • Belief that data can help prevent outbreaks​ • Concerned with protecting family and community​ • More support when purpose is clearly communicated​
	“If the goal is public safety and there are clear explanations about what is being done and why, then I think it’s a great way to use science to protect the population.”	
Education and communication	“Education is key—if you don’t tell people what’s happening in their own communities and how it affects them, they’ll feel left out or even threatened. Communication builds trust.”	• Desire for simple explanations of purpose and process​ • Trust relied on ongoing, honest communication​ • Many were unfamiliar with wastewater monitoring • Support linked to clear, transparent messaging​ • Openness increased with basic information​
	“This is my first time hearing about it. But it sounds like a good idea—if it’s explained well and backed up with science. People just need to understand what’s going on and why.”	

### Trust in Government and Authorities

Respondents expressed a range of views about trust in agencies managing WS. Many voiced confidence in local and state health departments and universities, citing their commitment to evidence-based practice, ethics, and responsiveness to community needs. Others expressed skepticism, raising concerns about transparency, surveillance, potential data misuse, and fears of political influence or excessive government control. Key concerns included uncertainty about who can access data, how data are used, and whether specific communities might be unfairly targeted. For many, distrust was shaped by previous negative experiences with public health programs or messaging. Trust was highest when respondents believed monitoring was transparent, locally led, and focused on public health outcomes.

### Effectiveness and Accuracy of Monitoring

Many respondents viewed WS as a valuable tool for tracking community health and providing early warnings about disease trends, especially when conducted by trusted institutions. Concerns included the validity of the testing methods, potential misinterpretation of results, and lack of transparency in data handling or use. Overall, support was strongest when respondents believed monitoring was scientifically rigorous, clearly explained, and overseen with integrity.

### Public Health Implications

Several respondents recognized WS as a valuable approach to protecting community health, especially for early outbreak detection and tracking public health trends. That support was often grounded in the belief that such efforts serve the public good and aim to prevent harm. Participants highlighted the importance of early detection. Confidence in the agencies conducting monitoring also emerged as a key theme, and some participants stated that they trust authorities to do the right thing. Although support was generally strong, a few respondents emphasized that their approval was conditional as long as WS was used for public health reasons. Overall, acceptance was highest when the purpose was clearly linked to community wellbeing and led by trusted institutions.

### Education and Communication

Many respondents emphasized the importance of clear communication and public education in building support for WS. Several indicated they were unfamiliar with the practice but expressed openness when given more information. Others stressed the need for accessible explanations and proactive outreach. Trust often hinged on clear and honest communication. Still, concerns about limited awareness remained, with some noting they did not know monitoring was occurring. Overall, respondents viewed education and communication as essential for fostering public understanding, transparency, and trust in WS efforts.

## Discussion

In this June 2024 nationwide survey, most respondents did not have concerns about WS for infectious diseases. However, some expressed limited or no awareness of WS and a desire for additional information and improved communication about findings from the initiatives.

Because large-scale use of WS is relatively new, few studies examining public perceptions of these initiatives are available. Results of several national surveys found that about half of respondents reported previous awareness of wastewater monitoring, but most were generally supportive of this public health strategy (*3*–*5*). Another survey found that 65% of participants (n = 516) knew little or nothing about WS and suggested the need for communication about how WS works, how findings are used, and protections against misuse (*6*). Those findings align closely with the results from our analysis and suggest a consistent pattern of support for WS while highlighting the opportunity for continued public education and communication about those efforts.

The findings from this survey highlight the trust that communities have in state and local public health and suggest that communities trust WS when they trust the institutions conducting it. They also suggest that, to further build and maintain trust, WS activities should remain transparent and focused on informing public health action and preventing community harm. The findings also highlight the opportunity for additional efforts to educate the public on why WS is used and how wastewater data are protected.

To engage community members, public health entities can share messages around individual health choices through trusted messengers like healthcare providers, community health workers, and local leaders. Public health entities should also build strong relationships with community and faith-based organizations to better reach local audiences and align communication strategies with the needs and preferences of the populations they serve (*7*–*9*).

The first limitation of this study is that it relied on a convenience sample and an internet-based, English-only survey; persons with limited internet access, less familiarity with online technology, or limited English proficiency might have been excluded. Therefore, the results are not representative of the entire population. In addition, because open-ended questions were optional, responses might not reflect the full range of perspectives on WS.

In conclusion, the survey’s findings highlighted opportunities for public health to use WS to build trust. Engaging communities through trusted messengers and organizations can enhance understanding and acceptance, and continued education and transparent communication could increase public awareness and strengthen confidence in public health initiatives. Together, those strategies can support effective individual and community responses to infectious disease threats.

Appendix Additional information on exploration of public perceptions of wastewater surveillance, United States, June 2024.
