# Supplementary material for: Exploration of Public Perceptions of Wastewater Surveillance, United States, June 2024
Source: Emerg Infect Dis. 2026 Aug;32(Suppl 1):S115–8. doi: 10.3201/eid3213.251494 (PMC13585668; doi:10.3201/eid3213.251494)
Supplement: Supplementary file 1 [file 25-1494-Techapp-s1.pdf]

Article DOI: https://doi.org/10.3201/eid3213.251494

EID cannot ensure accessibility for supplementary materials supplied by authors. Readers who have difficulty accessing supplementary content should contact the authors for assistance.

Exploration of Public Perceptions of Wastewater Surveillance, United States, June 2024

Appendix

Detailed Survey Methods

This PN View survey was conducted by Big Village (https://big-village.com/about), a research and analytics firm, among a sample of 2,024 U.S. adults 18 years of age and older. This survey was live on June 19–23, 2024.

PN View surveys are designed by Porter Novelli Public Services often with input from their clients. They are programmed and fielded by Big Village using opt-in panel members from Cint's Insight Exchange. Quota sampling, a non-probability sampling strategy where participants are selected based on pre-determined subgroups, is used to collect a nationwide sample of respondents. Responses are then weighted by gender, age, region, race/ethnicity, and education to mirror the demographic composition of the U.S. population using Current Population Survey proportions.

Data quality checks are incorporated during both sampling and survey administration. Cint uses a variety of tracking measures to confirm respondent identity and prevent duplicate responses. Big Village embeds quality control questions within each survey to identify and remove inattentive respondents.

Although Porter Novelli Public Services and Big Village are not subject to institutional review board review, they adhere to professional standards and codes of conduct set forth by ESOMAR (the European Society for Opinion and Marketing Research) and the Insights Association. Respondents are informed that their answers are being used for market research and

can refuse to answer any question at any time. Respondents receive an incentive ranging from $5-$10 for completing surveys through this platform. No personal identifiers are included in the resulting data files.

Respondents for each survey are selected from among those who have volunteered to participate in online polls. They may be subject to multiple sources of error, including, but not limited to sampling error, coverage error, error associated with nonresponse, error associated with question wording and response options, and post-survey weighting and adjustments.

Traditional response rates cannot be calculated because response links are made available to anyone in the study sample pool. To ensure rapid fielding, no effort is made to limit the number of “clicks” so quotas often fill before respondents have a chance to respond (Appendix Table).

Appendix Table. Details on survey clicks and completion

Details	Counts
Clicks	9,167
Completes	2,024
Incompletes	1,199
Terminate/No response	5,944

Survey Text

This Porter Novelli View survey was conducted by Big Village among a sample of 2,024 U.S. adults 18 years of age and older. This survey was live on June 19–23, 2024. The responses to question nine only are presented in this manuscript.

Demo1

How do you identify? **[Select one answer]**

1. Male

2. Female

3. Non-Gender Conforming - (including nonbinary, pangender, gender fluid, bigender, agender, transgender or other)

4. Prefer to self-identify **[Please specify]**

5. Prefer not to identify

Demo2

What is your age? [Enter a number. Programming note: Range is 5–99]

IF 18 YEARS OF AGE OR OLDER, CONTINUE.

ALL OTHERS TERMINATE

1. 18–20
2. 21–24
3. 25–29
4. 30–34
5. 35–39
6. 40–44
7. 45–49
8. 50–54
9. 55–59
10. 60–64
11. 65–69
12. 70–74
13. 75 or older

Demo3

What state do you live in? [Drop down menu with 50 states and Washington DC]

Northeast
Maine, New Hampshire, Vermont, Massachusetts, Rhode Island, Connecticut, New York, New Jersey, Pennsylvania
Midwest
Ohio, Indiana, Illinois, Michigan, Wisconsin, Minnesota, Iowa, Missouri, North Dakota, South Dakota, Nebraska, Kansas
South
Delaware, Maryland, District of Columbia, Virginia, West Virginia, North Carolina, South Carolina, Georgia, Florida, Kentucky, Tennessee, Alabama, Mississippi, Arkansas, Louisiana, Oklahoma, Texas
West

Montana, Idaho, Wyoming, Colorado, New Mexico, Arizona, Utah, Nevada, Washington, Oregon, California, Hawaii, Alaska

[ASK OF GEO STUDIES ONLY, UNLESS SPECIFICALLY REQUESTED]

Demo4A

In which of the following counties do you reside? [Select one answer]

DISPLAY QUALIFYING COUNTIES, AND NONE OF THESE. IF ANY OF QUALIFYING COUNTIES, CONTINUE. ALL OTHERS THANK AND TERMINATE

Demo4

What is your ZIP code? [Please enter 5-digit ZIP code, do not enter a range]

Demo19

Which of the following best describes the type of community you live in? **[Select one answer]**

1. Urban community
2. Suburban community
3. Rural community

Demo5

What is your marital status? [Select one answer]

1. Married
2. Living with a partner
3. Single and never been married
4. Separated
5. Divorced
6. Widowed

Demo6

Do you own or rent the dwelling in which you live, or do you live with others at no cost (i.e., parents, relatives, friends, etc.)? [Select one answer]

1. Own

2. Rent

3. Live with others at no cost

Demo7

Including yourself, how many adults 18 years of age and older are currently living in your household? [Enter a number. Programming note: Range is 1–10]

Demo8

Do you have any children under the age of 18 living at home? [Select one answer]

1. Yes

2. No

[ASK IF DEMO8 (01)]

Demo9

Can you please tell us how many children in each of the following age groups are living in your household? [Enter a number for each. Programming note: Range is 0–10. Respondent must give an answer to at least one of the ranges]

A. Children aged 6 or younger

B. Children aged 7–12

C. Children aged 13–17

Demo10

What is the highest level of formal education you have completed? [Select one answer]

1. Some schooling completed/No high school diploma

2. High school graduate or the equivalent (i.e., GED)

3. Some college credit, no degree

4. Associate degree/Technical school

5. Bachelor's degree

6. Master's degree

7. Professional/Doctorate degree

Demo11

Which of the following best describes your current employment status? [Select one answer]

1. Work full-time
2. Work part-time
3. Self-employed
4. Student
5. Homemaker
6. Retired
7. OMITTED
8. Unemployed and seeking work
9. Unemployed and NOT seeking work
10. Unable to work because of a disability

Demo12

Which of the following includes your <u>total</u> household income before taxes in 2023? [Select one answer]

1. Less than $25,000
2. $25,000-$29,999
3. $30,000-$34,999
4. $35,000-$39,999
5. $40,000-$49,999
6. $50,000-$59,999
7. $60,000-$74,999
8. $75,000-$99,999
9. $100,000-$124,999

10. $125,000-$149,999

11. $150,000-$199,999

12. $200,000 or more

Demo13

OMITTED

Demo14

What race do you consider yourself? [Select as many as apply]

1. African

2. African American/Black

3. Asian-American

4. Caribbean American

5. East Asian (Including Chinese, Japanese, Korean, Mongolian, Tibetan and Taiwanese)

6. Hispanic/Latinx

7. Indigenous American/First Nations (Including Native American/American Indian, Alaska Native, Pacific Islander, and Native Hawaiian)

8. Middle Eastern or North African

9. South Asian (including Bangladeshi, Bhutanese, Indian, Nepali, Pakistani and Sri Lankan)

10. Southeast Asian (Including Burmese, Cambodian, Filipino, Hmong, Indonesian, Laotian, Malaysian, Mien, Singaporean, Thai, and Vietnamese)

11. White

12. Not Listed **[Please specify]**

Demo1A

Do you identify as part of the LGBTQ+ community? **[Select one answer]**

1. Yes

2. No

3. Prefer not to say

Demo22

To help ensure we obtain answers from a diverse group of Americans, please select the items that describe you. **[Select as many as apply for 01–06. Randomize 01–06]**

1. You are deaf or have serious difficulty hearing

2. You are blind or have serious difficulty seeing even when wearing glasses

3. Because of a physical, mental or emotional condition, you have serious difficulty concentrating, remembering, or making decisions

4. You have difficulty walking or climbing stairs

5. You have difficulty dressing or bathing

6. You have difficulties doing errands alone such as visiting a doctor's office or shopping

7. None of these **[EXCLUSIVE]**

Demo20

In politics today, do you usually think of yourself as a Republican, a Democrat, an Independent, or something else? **[Select one answer]**

1. Republican

2. Democrat

3. Independent

4. Something else

5. Not sure

6. Decline to answer

[ASK IF DEMO20 (03–06)]

Demo21

As of today, do you lean more to the Republican party or the Democratic party? **[Select one answer]**

1. Republican

2. Democratic

3. Neither/Other

4. Decline to answer

On another subject . . .

W1 When you use the toilet, wash your hands, or wash your clothes, wastewater goes into the sewage system. Public health agencies test wastewater in communities to track the spread of public health threats. Have you heard of each of the following wastewater activities? **[Select one answer for each. Do not randomize A-C]**

1. Yes

2. No

3. Not sure

A. Wastewater being used to monitor for COVID-19

B. Wastewater being used to monitor for seasonal viruses like flu and RSV (respiratory syncytial virus)

C. Wastewater being used to monitor for mosquito-borne diseases like West Nile virus and dengue

W2 Have you seen wastewater monitoring data displays before, like these charts/graphs? **[Select as many as apply for 01–05. Do not randomize]**

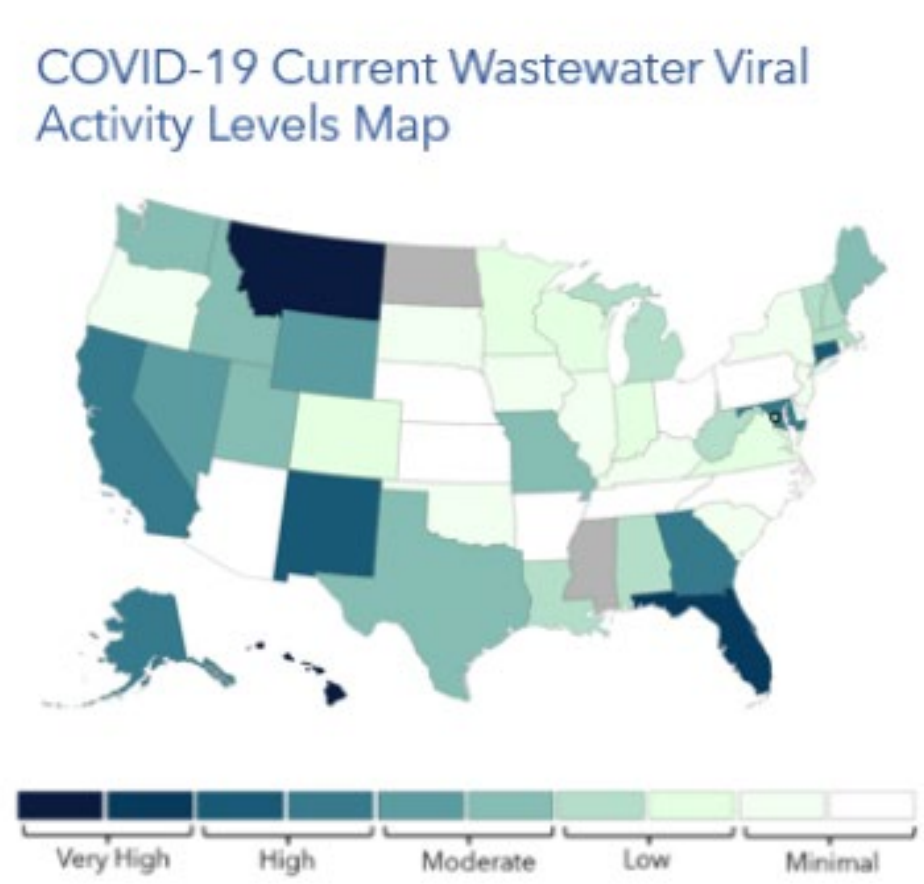

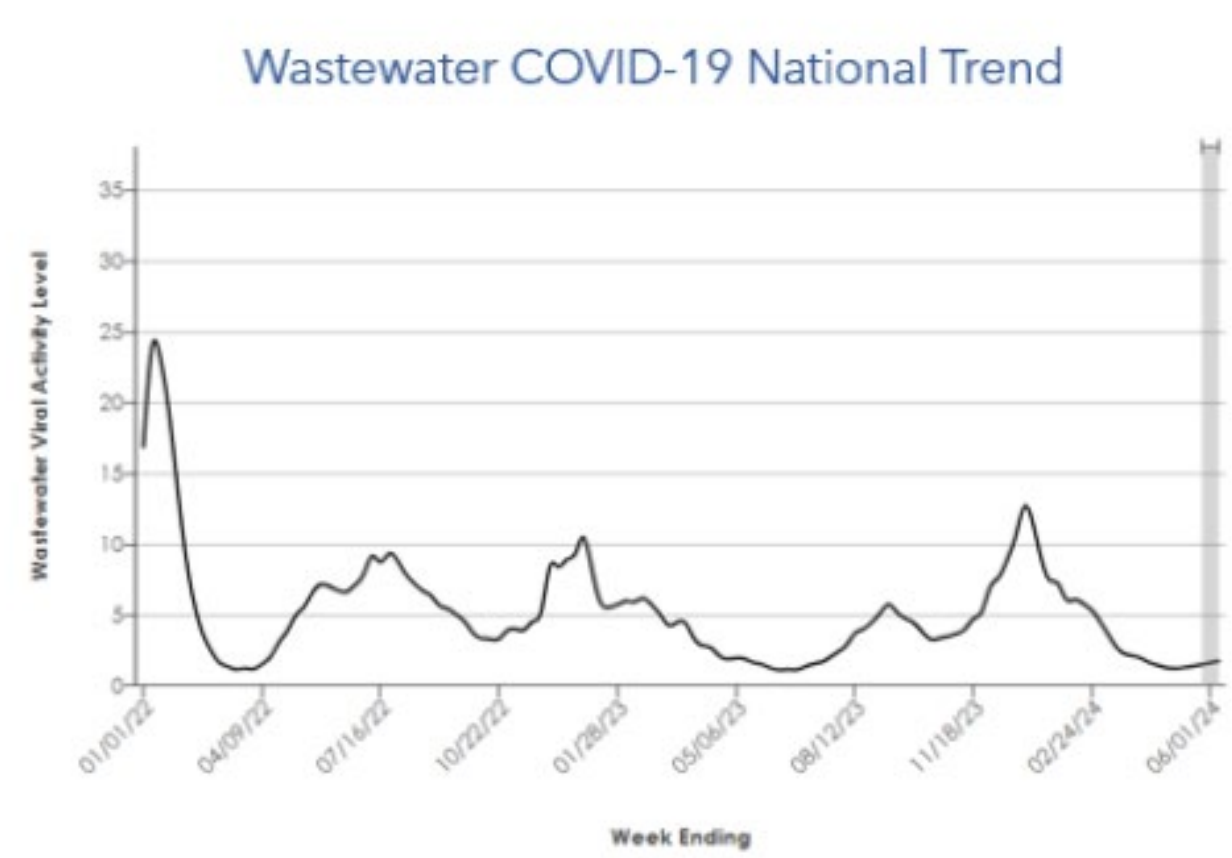

1. Yes, I saw wastewater monitoring data on the CDC Web site
2. Yes, I saw wastewater monitoring data on a Web site other than the CDC
3. Yes, I saw wastewater monitoring data on social media
4. Yes, I saw wastewater monitoring data on the news
5. Yes, I saw wastewater monitoring data from a different source **[Please specify]**
6. Yes, I saw wastewater monitoring data but don't remember where **[EXCLUSIVE]**
7. No, I have not seen wastewater monitoring data before **[EXCLUSIVE]**
8. I don't know **[EXCLUSIVE]**

W3 If wastewater data showed that a virus, like COVID-19 or flu, was spreading in your community, how likely would you be to take action to protect yourself? [Select one answer]

1. Very likely
2. Somewhat likely
3. Not too likely
4. Not likely at all

W4 What specific health behaviors would you consider engaging in to protect yourself if wastewater data showed that a virus like COVID-19 or flu was spreading in your area? **[Select as many as apply for 01–06. Randomize 01–05]**

1. Avoiding large gatherings
2. Wearing a mask in public indoor spaces
3. Washing hands more frequently
4. Getting a vaccine or booster
5. Sharing the information with my family or friends
6. Other **[Please specify]**
7. None **[EXCLUSIVE]**

W5 How comfortable are you with each of the following scenarios? **[Select one answer for each. Do not randomize A-C]**

1. Very uncomfortable
2. Somewhat uncomfortable
3. Neutral
4. Somewhat comfortable
5. Very comfortable

A. Wastewater being monitored in your community for respiratory diseases like COVID-19 and flu

B. Wastewater being monitored in your community for mosquito-borne diseases like West Nile virus and dengue

C. Wastewater being monitored in your community for opioids or other drugs

W6 Analyzing a community's wastewater might help to understand which drugs are used in the community, such as opioids. Opioid usage includes illegal and legal use of prescription drugs. If they find opioids in a community's wastewater, it can help inform public health efforts that help prevent overdoses. To what extent do you agree or disagree with the following statements? **[Select one answer for each. Do not randomize A-C]**

1. Strongly disagree
2. Somewhat disagree
3. Neither agree nor disagree
4. Somewhat agree
5. Strongly agree

A. Monitoring wastewater can help understand the level of opioid use in the community

B. The information obtained from wastewater monitoring about drugs should be used to guide public health actions

C. I trust that public health officials will use wastewater data only to improve the public's health

W7 How comfortable are you with each of the following **being used** to inform public health prevention activities? **[Select one answer for each. Do not randomize A-C]**

1. Very uncomfortable

2. Somewhat uncomfortable

3. Neutral

4. Somewhat comfortable

5. Very comfortable

A. Wastewater data on infectious diseases in my community

B. Wastewater data on infectious diseases in schools and universities

C. Wastewater data on diseases like COVID-19 in healthcare facilities

W8 How accurate do you think the following are? **[Select one answer for each. Do not randomize A-C]**

1. Not at all accurate

2. A little accurate

3. Mostly accurate

4. Completely accurate

5. Not sure

A. Information on the level of COVID-19 in my community's wastewater

B. Information on the level of mosquito-borne viruses in my community's wastewater

C. Information on the level of opioid drugs in my community's wastewater

W9 Do you have any concerns about wastewater monitoring in your community? Why or why not? **[Please type in your response]**
